# Modeling the synovium on a chip for studying the interactions between lymphatic vasculature and synoviocytes in rheumatoid arthritis

**DOI:** 10.1088/1758-5090/ae9348

**Published:** 2026-08-13

**Authors:** Samantha E Kraus, Issahy Cano, Soo Jin Kim, Esak Lee

**Affiliations:** 1 Nancy E. and Peter C. Meinig School of Biomedical Engineering, Cornell University, Ithaca, NY 14853, United States of America; 2 Department of Biomedical Sciences, College of Veterinary Medicine, Cornell University, Ithaca, NY 14853, United States of America

**Keywords:** rheumatoid arthritis, microfluidic chip, lymphatics, synovium

## Abstract

Rheumatoid arthritis (RA) is a chronic autoimmune inflammatory disorder that afflicts the synovial lining of joints and manifests in reduced range of motion, pain, swelling, and numerous other complications with no effective cure. In recent years, attention has shifted toward eradicating the source of synovial autoimmunity and inflammation, with a key focus on the synovial-draining lymphatics. However, very few *in vitro* models of the synovial microenvironment have been developed to date, and none yet include the synovial lymphatics. We therefore create a microfluidic chip device that models the synovial-draining lymphatics within the subintimal synovium microenvironment. Functional assays on our synovium-on-chip demonstrate increased lymphatic permeability and decreased drainage under RA inflammation compared to healthy controls, accompanied by increased lymphatic endothelial cell (LEC) junctional disruption and altered LEC phenotype following interaction with fibroblast-like synoviocytes. We identify overexpression of chitinase-3 like-protein-1 from RA patient-derived synoviocytes as a key target in inducing junctional loosening and lymphatic dysfunction, which is reversed in our microfluidic chip and in vivo mouse models by neutralizing antibody inhibition. Our novel synovium-on-chip model can provide a physiologically accurate representation of lymphatic drainage and activity under disease conditions, informing tissue engineering, drug testing, and high-throughput screening for RA.

## 1. Introduction

Rheumatoid arthritis (RA) is one of the most common chronic inflammatory joint disorders, affecting 0.5%–1% of the nearly 8 billion population worldwide with autoimmune cartilage degradation and synovial inflammation [1]. Symptoms manifest as overgrowth of macrophage-like synoviocytes (MLSs, or type A synoviocytes) and fibroblast-like synoviocytes (FLSs, or type B synoviocytes) that comprise the synovial membrane lining the joint capsule, leading to hyperplasia and eventually compromising synovial fluid lubrication, resulting in inflammatory joint swelling and articular cartilage degradation [2]. While the precise etiology of the disease is hypothesized to be a combination of environmental, hormonal, and genetic factors, the condition stems from a failure of the adaptive immune system’s self-tolerance, causing autoreactive T cells, autoantibodies, and inflammatory macrophages to infiltrate the synovium and attract degradative enzymes that damage the synovial tissue and cartilage. The inflammatory cascade is primarily driven by macrophage- and fibroblast-derived cytokines, including IL-1, IL-6, IL-15, IL-17, IL-18, granulocyte-macrophage colony-stimulating factor (GM-CSF), and tumor necrosis factor (TNF-*α*) [3]. Many of these inflammatory cytokines act synergistically to promote inflammation; for example, IL-17 produced by Th17 helper T cells and mast cells induces TNF-*α* and matrix metalloproteinase (MMP) secretion from macrophages, as well as adhesion molecule expression in endothelial cells [4–6]. The most common treatments for RA clinically include disease-modifying antirheumatic drugs (DMARDs) such as methotrexate and biological agents, and cytokine inhibitors such as etanercept [7–9]. However, the complexity of the inflammatory response in RA and heterogeneity amongst patients has rendered drug discovery difficult, and many patients become refractory to current treatments or suffer increased risk of infection due to systemic immunosuppression [10, 11]. Thus, new areas for therapeutic targeting of RA are being explored, including the role of the synovial lymphatics.

The two primary functions of the lymphatic system are maintaining interstitial fluid balance and transporting immune cells [12]. The initial lymphatic vessels (LVs), consisting of a single layer of lymphatic endothelial cells (LECs) with highly permeably ‘button-like’ cell–cell junctions, allow uptake of fluid and macromolecules from the interstitial tissue via the primary valves [13, 14], and later converge into collecting vessels with tight ‘zipper-like’ cell-cell junctions and lymphatic muscle cells that contract fluid unidirectionally to the lymph nodes and eventually systemic circulation [15]. Additionally, the lymphatic system plays an integral role in the adaptive immune response, as antigen presenting cells, such as dendritic cells (DCs), from the periphery travel through the afferent lymphatics to the lymph nodes, where T and B lymphocytes are activated by a specific foreign antigen and are transported via the efferent lymphatics to sites of inflammation to mount an immune response [16, 17]. Due to the lymphatic system’s inherent role in interstitial fluid buildup and immune cell trafficking, dysfunction of the lymphatics is implicated in numerous maladies, including autoimmune and rheumatic diseases [18].

Studies using near-infrared lymphangiography and magnetic resonance imaging in patients with RA [19, 20] have identified two major phases of lymphatic changes in the pathogenesis of the disease: the expansion phase and the collapsed phase. In the expansion phase, initial pre-arthritic inflammation causes increased lymphatic contractions and rapid lymphatic drainage to remove inflammatory cells and cellular debris [21, 22]. This process is accompanied by the rapid migration of immune cells, such as DCs, to the lymphatic collecting vessels via CC chemokine receptor 7 and integrin-mediated binding mechanisms, increased lymphangiogenesis, and swelling of the draining lymph nodes [23]. The removal of inflammatory cells and catabolic factors during the expansion phase damages the LECs/lymphatic smooth muscle cells (LMCs) (lymphatic muscle cells) of the afferent lymphatics, leading to the symptomatic collapsed phase. During this phase, the collapse of the synovial lymphatics leads to impaired lymphatic clearance, increased vessel leakiness, decreased contractions, and stasis of inflammatory fluid in the joint [24, 25].

Given the remarkable changes to the synovial lymphatics in RA, a suitable model for the synovium and associated vasculature under healthy and diseased conditions would provide a platform for mechanistic studies and clinical drug testing. Non-human in vivo models require the injection of soluble agents or genetic manipulation to develop autoimmune diseases and often can only recapitulate select aspects of the disease, such as articular cartilage erosion, failing to reflect the full disease pathophysiology [26–28]. Additionally, the inherent complexity and variability of RA render it difficult to decouple the individual biological factors contributing to disease. Therefore, a reliable *in vitro* model could provide a high-throughput, fiscally feasible method of examining the interactions between isolated factors that contribute to synovial inflammation in RA, as reviewed in detail previously [29]. The versatile organ-on-chip platform offers high tunability of fluid shear stress and cell micropatterning, and enables consistent monitoring of pH, temperature, and other culture conditions, while also allowing the incorporation of patient-specific cells for personalized therapy [30, 31]. Current *in vitro* models of the synovium microenvironment have focused on certain aspects, such as the subchondral bone and articular cartilage interface [32], or only incorporated isolated synovial organoids [33] or blood endothelial vessels [34, 35]. Some more advanced models have successfully incorporated both FLS and MLS cells in tri-culture with blood endothelial cells [36] or ventured into high-throughput testing of patient-specific responses in synovial and endothelial cell spheroid models [37].

We therefore created a synovium-on-chip microfluidic device that for the first time models the synovial tissue and associated LVs *in vitro*, providing a unique, isolated platform to examine the effect of synovial fibroblasts on lymphatic structure and function in RA.

## 2. Materials and methods

### 2.1. Cell culture

Primary human dermal LECs, isolated from dermal tissue of neonatal donors, were kindly provided by Dr Young Kwon Hong (University of Southern California). These cells have been characterized using several lymphatic endothelial markers in Dr Hong’s lab [38, 39] and verified to express the LEC-specific marker Prox1, which colocalizes with DAPI, as shown previously [40]. LECs were cultured in EGM-2 MV media (Lonza, Switzerland) and used in passages 6-12. Primary human FLSs, isolated from both healthy donors and donors with RA (FLS-RAs), were obtained from Cell Applications, Inc. FLSs were cultured in HFLS Growth Medium (Cell Applications, San Diego, CA) and used in passages 4–9. All cell types were maintained in standard tissue culture incubators at 37 °C, 95% humidity, and 5% CO^2^.

### 2.2. Synoviocyte gelation and characterization

To characterize FLSs and FLS-RAs, both cell types were seeded at 0.5 million cells/ml in 2D cell culture 6-well plates, cultured for 5–6 d, and then fixed and stained for DAPI (Sigma, St. Louis, MO, 1:500), phalloidin (Life Technologies, Carlsbad, CA, 1:200), CDH11 (Invitrogen, Waltham, MA, 1:200), hyaluronic acid (HA) (Amsbio, Cambridge, MA, 1:100), CD90 (Life Technologies, 1:100), and PRG4 (Abcam, Cambridge, UK, 1:100) similar to the procedures used for microfluidic chips described later. Confocal images were taken of the cells as described using a SP8 confocal microscope (Leica) with a 10x objective, and equal-sized images were thresholded to obtain actin and CDH11 signal, normalized to cell number, and quantified by DAPI signal. For optimization of 3D culture of FLSs and FLS-RAs, both cell types were seeded at 0.5 million cells/ml in a collagen and HA composite hydrogel on glass coverslips in 4-well plates using methods described in the following section. Synoviocyte-seeded gels were cultured for 5–6 d, then fixed, stained, and imaged as described with DAPI and phalloidin. Images of equal size for each group were obtained, and the roundness index (RI) was calculated for all cells within the area of interest using ImageJ according to the following equation:

\begin{equation*}{\text{Roundness Index }}\left( {{\mathrm{RI}}} \right) = \frac{{{\text{cell area}}}}{{\pi \times {\mathrm{2}}\left( {{\mathrm{fere}}{{\mathrm{t}}^{\prime}}{\text{s diameter/2}}} \right)}}.\end{equation*}

### 2.3. Microfluidic chip fabrication

Microfluidic devices were fabricated using soft lithography, as previously described [41–44]. Briefly, the human LV chip consisted of a polydimethylsiloxane (PDMS) housing with two channels. The PDMS (Sylgard 184, Dow Corning, Midland, MI) was prepared by mixing a 10:1 base-to-curing agent ratio and pouring it into silicon master molds for overnight curing at 80 °C. After removal from the molds, the PDMS was treated with plasma etching and attached to a cover glass. The devices were then treated with 0.01% poly-L-lysine (Sigma) for 1 h, then with 1% glutaraldehyde for 30 min, and finally rinsed thoroughly overnight. The channels of the device were prepared by inserting sterilized, 0.25 mm-diameter steel acupuncture needles coated with bovine serum albumin (BSA). A collagen and hyaluronic acid composite hydrogel encapsulating healthy or RA patient-derived FLSs was pipetted into the devices to form a cohesive extracellular matrix (ECM) and polymerized for 50 min at 37 °C. The ECM consisted of 2.5 mg ml^−1^ collagen 1 (Corning), 0.8 mg ml^−1^ thiol-treated hyaluronic acid (HA, HyStem kit from Advanced Biomatrix, Carlsbad, CA), 0.1 mg ml^−1^ human plasma fibronectin (Sigma-Aldrich), 10× phosphate-buffered saline (PBS, Gibco, Waltham, MA), 1 N sodium hydroxide (NaOH, Electron Microscopy Sciences, Hatfield, PA), and HFLS Growth Media containing healthy or RA patient-derived FLSs with a final concentration of 0.2 million cells per ml. For our purposes, the collagen 1 ECM is chosen for support of LEC growth as used in past studies from our lab [44] and based on behavior in vivo [45]. Hyaluronic acid (HA)/hyaluronan is the most abundant component of synovium ECM at a concentration of 0.8 mg ml^−1^ per extrafibrillar space. Lubrication and fluid retention need to provide mechanical absorption within the joint [46, 47] hence, their use at physiologically relevant concentrations in the fabricated ECM, along with fibronectin, a glycoprotein that facilitates cell-ECM interactions [48]. Rheological studies on our collagen and HA composite hydrogel determined that the material exhibits a storage modulus of 121.3 Pa, a loss modulus of 30.7 Pa, and a complex viscosity of 198.3 Pa*s at a 0.1 Hz oscillation frequency and 3% strain rate, similar to GelMA hydrogels with storage modulus of 125.5-190.3 Pa used previously to culture FLSs to create biomimetic synovial tissue [49]. Ensuing overnight media washing and needle removal, LECs were added to one of the two channels by resuspending the cells at 1.25 × 10^6^ cells ml^−1^ in LEC media and introducing the cell solution to a single channel to allow cell adhesion to the 3D collagen matrix for 10 min before washing out excess cells with growth medium. The devices were incubated for 1–2 d on a rocking platform in the tissue culture incubator, replenishing culture media daily. To determine the shear stress imparted by a rocker, we assumed that the culture medium is a Newtonian fluid in which the viscous stresses arising from its flow at every point are linearly correlated with the local strain rate, as detailed in our previous study [44]. We determined that the shear stress created by the rocker, 3.5–4.5 dyne cm^−2^ falls within the physiological range of 4–12 dyne cm^−2^ seen in rat mesenteric pre-nodal lymphatics [50]. For stimulation of FLSs with inflammatory cytokines to mimic the RA environment, 50 ng ml^−1^ IL-17 (Sigma) and 0.5 ng ml^−1^ TNF-*α* (PeproTech, Cranbury, NJ) were added to the EGM-2 MV media replenished in the device reservoirs 24 h before drainage, permeability, lymphangiogenesis, or immune cell migration assays and then washed out with blank EGM-2 MV media.

### 2.4. Lymphatic drainage

For measuring lymphatic drainage (fluid uptake) in the microfluidic chip devices, fluorescently labeled dextran (70 kDa, FITC, Life Technologies) was mixed into the EGM-2 MV media solution at a dilution of 1:1000 and added to the reservoirs on the opposite side of the lymphatic vascular channel to establish an interstitial fluid pressure gradient across the collagen 1 hydrogel with embedded FLSs toward the LV. After allowing the fluid to reach equilibrium over 16 h, the media was collected from reservoirs on both the vascular and avascular sides, and the samples were weighed to determine the volume of fluid collected, using the known density of the nanoparticle solution. The concentration of nanoparticles in the collected samples was then determined by fluorescence spectroscopy using a SpectraMax M2 Microplate Reader (Molecular Devices, San Jose, CA) and SoftMax® Pro 7 Software, with a standard curve of known concentrations in the medium. The degree of nanoparticle drainage was then determined as the percentage of total nanoparticles loaded that were drained to the lymphatic vascular channel.

### 2.5. Lymphatic permeability

Lymphatic permeability (fluid leakage) in microfluidic devices was measured as described previously [41, 42, 51]. Briefly, fluorescently labeled dextran (70 kDa, FITC, Life Technologies) was mixed into the media solution (dextran concentration: 25 *µ*g ml)^−1^, and 50 *μ*l of the dextran solution was added to one reservoir connected to the other reservoir through the lymphatic vascular channel. This initial hydrostatic pressure allows fluid to fill the vessel lumen. We imaged the vessel area (in a 10× field) sufficiently far from the dextran injection site to minimize fluctuations in the data caused by the initial loading pressure. We imaged dextran diffusion into the collagen matrix for 5 min after injection, acquiring images every 5 s with an SP8 confocal microscope (Leica, Germany). We adopted the previously developed automated MATLAB code, which, in summary, determines a linear fit of the integrated fluorescence signal in the interstitium (gel) over time and uses the slope of that fit as the diffusive permeability (cm s^−1^) to quantify permeability from time-lapse images [41, 42, 51].

### 2.6. Lymphangiogenesis assay

For lymphangiogenic studies, engineered LVs were cultured in one channel of the aforementioned microfluidic devices, with the distance between adjacent channels reduced from 5 mm to 1 mm to enable paracrine interactions between the two cell types in close proximity. The adjacent channel was seeded with a cell suspension of healthy or RA patient-derived FLS (0.75 million cells/ml). The devices were then incubated for at least 6 d to allow for lymphangiogenic sprouting before fixation and imaging.

### 2.7. Immune cell migration assay

For examination of immune cell dynamics within the synovium-on-chip, cryopreserved human peripheral blood mononuclear cells (PBMCs) (StemCell Technologies, Vancouver, Canada) were obtained, thawed, and resuspended for cell culture according to the manufacturer’s instructions, then labeled with CellTracker™ Red CMTPX Dye (Invitrogen) according to the kit’s instructions. The chips fabricated for immune cell studies were identical to those previously described, except that the ECM was replaced with a fibrin-collagen composite hydrogel, which is more amenable to immune cell migration in *in vitro* microfluidic culture [52]. The fibrin gel was created using methods similar to those by Chen *et al* [52]. Briefly, 30 mg of bovine fibrinogen (Sigma-Aldrich) was dissolved in 5 ml of sterile PBS at 37 °C on a rocking platform. The 6 mg ml^−1^ fibrinogen solution was mixed with a 1 mg ml^−1^ collagen 1 hydrogel solution formulated using the methods described previously at a 3:1 ratio to create the fibrinogen-collagen composite hydrogel, which was aliquoted and combined with thrombin solution (Sigma-Aldrich) at a ratio of 4 U thrombin to 10 mg of fibrinogen to form a polymerized fibrin gel encapsulating of healthy or RA patient-derived FLSs. To examine immune cell entry, a suspension of PBMCs (0.05 million cells/ml) was introduced to the reservoirs on the opposite side of the lymphatic vascular channel to establish an interstitial fluid pressure gradient towards the lymphatic channel.

### 2.8. Flow cytometry analysis

For flow cytometry characterization of immune cells used within the migration assays, cryopreserved human PBMCs were suspended as single cells, diluted to 1 × 10^6^ cells/100 *µ*l, and stained with Zombie Red Fixable Viability Kit (BioLegend, San Diego, CA, 1:500) for 20 min at 4 °C. Live/dead stained samples were fixed in the Fixation Buffer (BioLegend) and stored in the Cell Staining Buffer (BioLegend) at 4 °C. When all the samples were ready for staining, 100 *µ*l of each sample was placed in a Falcon® 5 ml Round Bottom Polystyrene Test Tube, pelleted at 300 × g for 5 min, and blocked with anti-mouse CD16/32 (BioLegend, 1:100) in the staining buffer on ice for 15 min. Primary antibodies, including BD Horizon™ BB515 mouse anti-human CD3 (BD Biosciences, Franklin Lakes, NJ, 1:20), PE/Fire™ 700 anti-human CD19 (BioLegend, 1:20), and Brilliant Violet 605™ anti-human CD14 (BioLegend, 1:20), were diluted and added to the samples and incubated on ice for 30 min in the dark. After staining, cells were washed and resuspended in 300 *µ*l of the staining buffer and analyzed on an Attune NxT Flow Cytometer (ThermoFisher) at the Cornell Institute of Biotechnology Flow Cytometry Core. Data were analyzed in FlowJo 11 (BD Biosciences).

### 2.9. Cytokine analysis

For cytokine analysis, media were collected from LECs, healthy FLSs, FLSs stimulated with inflammatory cytokines IL-17 and TNF-*α*, and RA patient-derived FLSs cultured in 2D, and an ELISA immunoassay was performed using the Proteome Profiler Human XL Cytokine Array Kit (R&D Systems, Minneapolis, MN) to screen for relative secretion levels of 105 human cytokines per the manufacturer’s instructions.

### 2.10. Chitinase-3 like-protein-1 (CHI3L1) protein and neutralizing antibody treatment

To test the effects of soluble CHI3L1 on LECs, 10 *μ*g ml^−1^ recombinant human CHI3L1 protein (R&D Systems) was added to the EGM-2 MV media replenished in the device reservoirs 24 h prior to drainage, permeability, or lymphangiogenesis assays and then washed out with blank EGM-2 MV media. Similarly, for experiments that tested the efficacy of inhibiting CHI3L1, 10 *μ*g ml^−1^ of Anti-YKL-40 [N-AY], Mouse IgG2a, Kappa (Absolute Antibody, Redcar, UK) was added to the EGM-2 MV media replenished in the device reservoirs 24 h before drainage, permeability, lymphangiogenesis, or immune cell migration assays, and then washed out with blank EGM-2 MV media. For in vivo studies with CHI3L1 inhibition, 200 *μ*g of Anti-YKL-40 (4 × 10^3^-F2), Mouse IgG2a, Kappa, or isotype control Anti-Fluorescein [4-4-20 (enhanced)], Mouse IgG2a, Kappa (Absolute Antibody) was injected intraperitoneally per mouse weekly from week 3 onward.

### 2.11. Western blot analysis

Confluent LECs grown on tissue culture-treated 6 well plates (Greiner, Monroe, NC) were starved for 24 h in basal media EBM2 (Lonza) before treatment with combinations of VEGFA (20 ng ml^−1^, PeproTech), CHI3L1 (10 *μ*g ml^−1^, R&D Systems), and CHI3L1-IN-1 inhibitor (10 *μ*M, MedChemExpress, Monmouth Junction, NJ) as well as sodium orthovanadate (200 mM, Sigma) to preserve phosphorylation. Cells were rinsed in cold PBS (Gibco) supplemented with 200 mM sodium orthovanadate, then lysed with Laemmli SDS-Sample Buffer (6X, Non-Reducing) (Boston Bioproducts, Inc., Milford, MA) diluted 1:3 in Pierce™ IP Lysis Buffer (Thermo Fisher, Waltham, MA) with 2× protease and phosphatase inhibitors: PhosSTOP™ (Sigma), cOmplete™ ULTRA Tablets, Mini, EASYpack Protease Inhibitor Cocktail (Sigma). Cell lysates were collected with a cell scraper, incubated on ice for 20 min, boiled at 95 °C for 8 min, and centrifuged at 10 000 × *g* for 2 min at 20 °C. The lysate proteins were fractionated by SDS-PAGE using Mini-PROTEAN® TGX™ Precast Gels 4%–20% (Bio-Rad, Hercules, CA), 1× Tris/Glycine/SDS running buffer (Bio-Rad) in the Mini-PROTEAN® Tetra System (Bio-Rad) at 120 V. The fractionated proteins in the gel were transferred to a nitrocellulose membrane using Trans-Blot® Turbo™ Mini Nitrocellulose Transfer Packs (Bio-Rad) and the Trans-Blot® Turbo™ Transfer Starter System (Bio-Rad). After protein transfer, the nitrocellulose membrane was rinsed with 1× PBS (Gibco) and blocked with EveryBlot Blocking Buffer (Bio-Rad) overnight at 4 °C. Primary antibodies and secondary antibodies (both diluted in EveryBlot Blocking Buffer) included anti-VEGF Receptor 2 (55B11) antibody (Cell Signaling, 1:1000), anti-Phospho-VEGF Receptor 2 (Tyr1175) antibody (Cell Signaling, 1:1000), anti-VE-cadherin (D87F2) (Cell Signaling, 1:1000), anti-Phospho-VE-cadherin (Tyr658) (Affinity Biosciences, Cincinnati, OH, 1:1000), anti-GAPDH antibody (Cell Signaling, 1:1000), and HRP-linked anti-rabbit IgG antibody (Cell Signaling, 1:1000). The protein bands were visualized by 4 min membrane exposure to Clarity Western ECL Substrate (Bio-Rad), and the chemiluminescent HRP was detected by the Amersham Imager 680 (GE Healthcare, Chicago, IL). The Western blot images were adjusted for brightness and contrast using ImageJ.

### 2.12. siRNA knockdown and RT-qPCR analysis

For siRNA transfection, RA patient-derived FLS cells were seeded in 12-well plates and incubated for 24 h. Control (scrambled) siRNA or CHI3L1 siRNA (Thermo Fisher, 4392420) was mixed with Lipofectamine RNAiMAX Transfection Reagent (Invitrogen). The siRNA-reagent complex was added to the cell culture to achieve a final siRNA concentration of 20 nM. Gene expression analysis was then performed using FLS-RA cells 48 h after the transfection. Total RNA was then isolated from the FLS-RA cells using the RNeasy Kit (Qiagen, Hilden, Germany) per the manufacturer’s instructions, and RNA purity was verified by measuring the 260:280 ratio. Reverse transcription was performed using the qScript Ultra SuperMix (Quantabio), followed by qPCR using PowerTrack SYBR Green Master Mix (Thermo Fisher) on a QuantStudio Pro 7 system (Biotechnology Resource Center, Cornell University). Primer sequences used for target genes are detailed in the supplementary materials (supplementary table 1).

### 2.13. Immunofluorescence staining and imaging in microfluidics

For immunofluorescent staining and imaging, microfluidic devices were fixed with 4% paraformaldehyde (Electron Microscopy Sciences). Fixed devices were permeated with PBST (0.3% Triton-X in PBS), then blocked with 3% BSA in PBS overnight at 4 °C. Primary antibodies were incubated overnight at 4 °C in a blocking buffer and then washed with PBS. The primary antibodies utilized in the various studies included VE-cadherin (Santa Cruz Biotechnology, Dallas, TX, 1:100), cadherin-11 (Invitrogen, 1:200), and CD31 (Dako, Santa Clara, CA, 1:100). Secondary antibodies (all from Invitrogen, 1:500), phalloidin (Life Technologies, 1:200), and DAPI (Sigma, 1:500) were subsequently incubated in a blocking buffer overnight at 4 °C in the dark and washed with PBS once more. Confocal images were acquired with an SP8 confocal microscope (Leica) with a 40× objective. The obtained fluorescent images were *z*-stacked and adjusted for brightness and contrast using ImageJ, then analyzed for fluorescent signal intensity of various channels [53, 54].

### 2.14. Animal studies

RA was modeled in mice using the standard collagen-induced arthritis (CIA) model, as described in Springer Protocols [55]. In summary, female 8 week-old C57BL/6 J mice (The Jackson Laboratory) were injected at the base of the tail with a homogenized emulsion of chicken type II collagen (Sigma) and Complete Freund’s Adjuvant (CFA), CFA (Chondrex Inc. Woodinville, WA), followed by a boost injection after three weeks. Control mice were given an injection of PBS. Twice weekly, mouse paw swelling was examined, and a clinical arthritis score was given on a scale of 0–3 [56] to ensure the development of polyarthritis within the paw joints. After 8 weeks from the first injection, mice were euthanized and sacrificed, and the mouse paws, blood, and inguinal and popliteal lymph nodes were collected for analysis and fixed in 4% formalin. The presence of anti-type II collagen IgG antibodies, indicative of autoimmune disease activity, was measured in mouse serum collected by cardiac puncture using a Mouse Anti-Mouse Type II Collagen IgG Antibody Assay Kit (Chondrex) per the manufacturer’s instructions. Mouse paws were decalcified in a solution of EDTA (Sigma-Aldrich) balanced for pH with sodium hydroxide (NaOH) pellets (Sigma-Aldrich) and hydrochloric acid (HCL), then embedded in Tissue-Tek® O.C.T. Compound and sectioned into 5 *μ*m sagittal slices for staining with a H&E Staining Kit (Hematoxylin and Eosin) (Abcam) per the manufacturer’s instructions. For immunofluorescent staining, sections were blocked with 10% BSA in PBST, then stained with primary antibodies overnight at 4 °C, followed by secondary antibodies overnight at 4 °C in 1% BSA. Primary antibodies included CD31 (BD Biosciences, Franklin Lakes, New Jersey, 1:200) and LYVE-1 (Novus, Centennial, CO, 1:200), and secondary antibodies included corresponding Alexa Fluor dyes (all from Invitrogen, 1:1000). For lymphatic drainage assays, fluorescently labeled dextran (70 kDa, Oregon Green^TM^, Invitrogen) was injected into the hind footpads of mice and the draining popliteal lymph nodes and collected 30–60 min afterward. The lymph nodes were digested, and the fluorescence intensity of dextran was measured by fluorescence spectroscopy using a SpectraMax M2 Microplate Reader and SoftMax® Pro 7 Software.

### 2.15. Statistics

Independent two-sample populations were compared using unpaired two-sample *t*-tests, assuming a normal distribution. For group analyses, one-way ANOVAs with Tukey’s HSD (Honestly Significant Difference) tests were used to compare the mean values. All *P* values were two-sided, and *P < 0.05 were considered statistically significant. Statistical analyses were performed using GraphPad Prism 9. All data points on the graphs represent average values, and error bars depict the standard error of the mean.

## 3. Results

### 3.1. Modeling the synovial subintima and associated lymphatics *in vitro* under healthy and RA disease conditions

**Figure 1.**
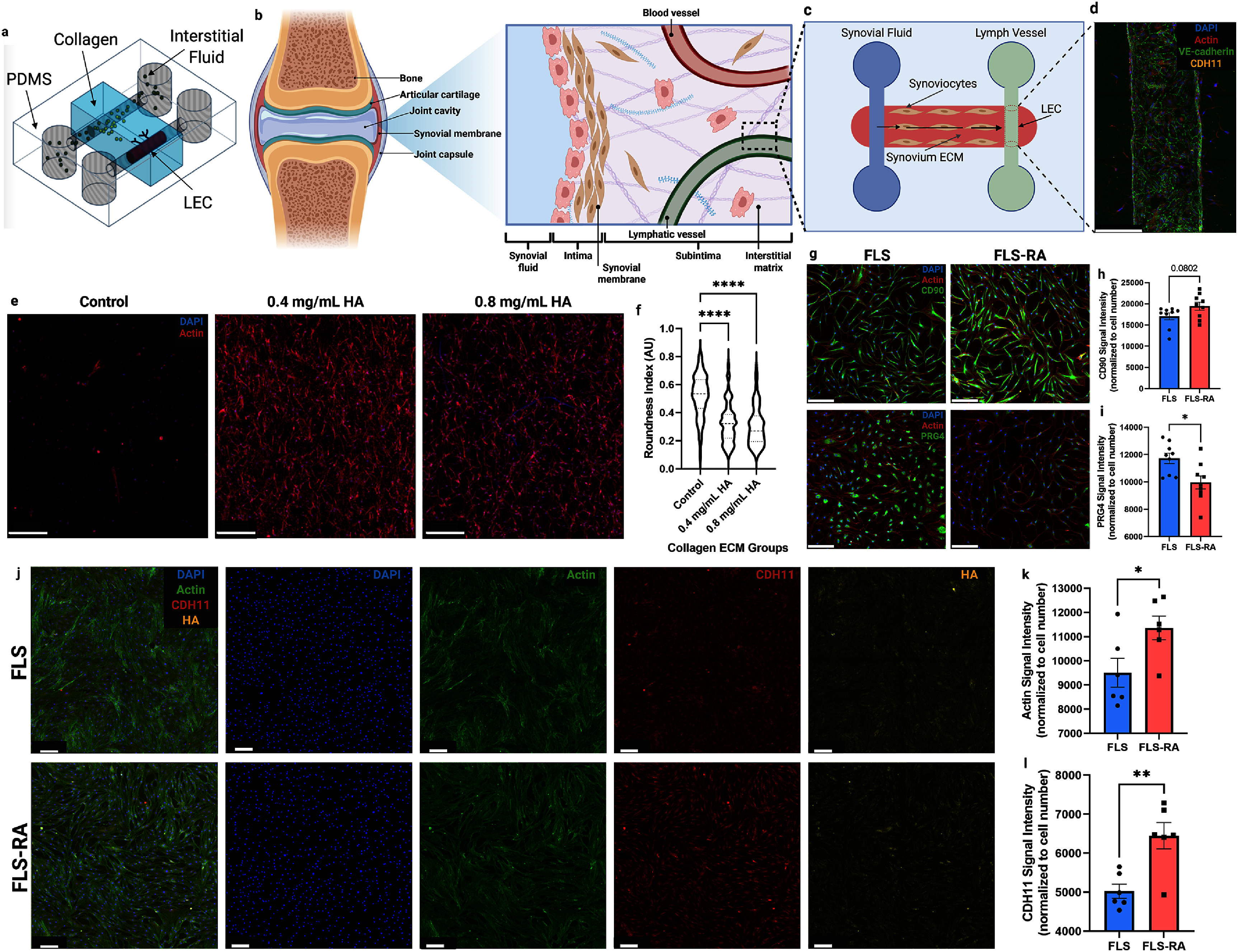
Synovium-on-chip models synovial subintima and associated lymphatics *in vitro*. (a) A basic schematic of the organotypic 3D synovial lymphatic vessel model with a channel of lymphatic endothelial cells (LECs) embedded in a biomimetic collagen 1 matrix and an acellular channel to induce interstitial fluid flow. (b) The human synovial joint consists of bone covered in articular cartilage suspended within a joint cavity containing synovial fluid, all encompassed within the joint capsule. The synovial membrane, or synovium, lines the inner surface of the joint capsule and consists of an intimal lining layer, or intima, and a sublining layer, or subintima. (c) The synovium-on-chip recapitulates a lymphatic vessel within the subintima extracellular matrix (ECM) embedded with FLSs, with the acellular channel allowing synovial fluid or media to induce interstitial fluid flow toward the lymphatic vessel. (d) An engineered lymphatic vessel stained with DAPI, phalloidin (actin), VE-cadherin (adherens junctions), and CDH11 (FLS marker). Scale bar = 200 *μ*m. (e) Representative images of 3D culture of FLSs seeded at the same concentration in collagen 1 hydrogel with hyaluronic acid (HA). Cells were stained with DAPI and phalloidin (actin). Scale bar = 400 *μ*m. (f) Violin plot of the roundness index (RI) of FLSs in each collagen ECM group. ****p < 0.0001; One-way ANOVA with Tukey’s HSD tests. (g) Images of 2D culture of healthy fibroblast-like synoviocytes (FLS) and RA patient-derived fibroblast-like synoviocytes (FLS-RA) stained with DAPI, phalloidin (actin), and CD90 (subintima FLS marker) or PRG4 (intima FLS maker). Scale bar = 200 *μ*m. Quantification of (h) CD90 and (i) PRG4 signal intensity normalized to cell number between FLS and FLS-RA. *p = 0.0107; two-tailed paired student *t-*test. (j) Images of 2D culture of healthy fibroblast-like synoviocytes (FLS) and RA patient-derived fibroblast-like synoviocytes (FLS-RA) stained with DAPI, phalloidin (actin), CDH11 (FLS marker), and HA (secreted by FLS). Scale bar = 200 *μ*m. Quantification of (k) actin and (l) CDH11 signal intensity normalized to cell number between FLS and FLS-RA. *p = 0.0365, **p = 0.0041; two-tailed paired student *t*-test, *n* shown as individual data points for each group. Data are expressed as mean ± S.E.M.

Our microfluidic model, previously used in our lab to examine LV behavior *in vitro* [40, 44], served as the basis for a synovium-on-chip. Briefly, our PDMS-based LV-on-chip comprises two hollow cylindrical channels that are completely embedded in a 3D collagen 1 matrix (figure 1(a)). One hollow channel is seeded with human dermal LECs to form an engineered LV, which is cultured for 1–2 d under luminal shear flow of 3.5–4.5 dyne cm^−2^ on a rocking platform in a tissue culture incubator until full LEC coverage is reached. Cell media is introduced into circular reservoirs directly connected to the LEC channel, or into reservoirs connected to the empty channel, to simulate luminal or interstitial fluid transport, respectively.

In the native human synovial joint, bone covered in articular cartilage is suspended within a joint cavity containing synovial fluid rich in molecules such as hyaluronic acid and lubricin to provide lubrication and shock absorption (figure 1(b)). The synovial membrane, or synovium, lines the inner surface of the joint capsule and consists of an intimal lining layer, or intima, about 1–3 cells thick with FLSs and a sublining layer, or subintima, which is relatively acellular, containing FLSs, MLSs, fat cells, blood vessels, and synovial-draining LVs [57]. The synovium-on-chip adapts the standard LV microfluidic chip design, but specifically aims to mimic a LV within the subintima ECM, recapitulated with FLSs embedded within the collagen ECM. The acellular channel allows media to induce interstitial fluid flow toward the LV, mimicking synovial drainage (figure 1(c)). Immunofluorescent staining of the engineered LV within the chip reveals expression of VE-cadherin, an endothelial cell adherens junction marker, and cadherin-11 (CDH11), an FLS marker surrounding the vessel (figure 1(d)).

To optimize the collagen 1 ECM and render it biomimetic for FLS culture, FLSs were seeded in 3D collagen 1 hydrogel with varying concentrations of hyaluronic acid (HA) (figure 1(e)). Hyaluronic acid (HA)/hyaluronan is the most abundant component of synovium ECM at a concentration of 0.8 mg ml^−1^ per extrafibrillar space and is essential for lubrication and fluid retention to provide mechanical absorption within the joint [46, 47], hence its use at a physiologically relevant concentration in the fabricated ECM. The inclusion of HA within the ECM significantly reduced the RI of FLSs (figure 1(f)), thereby allowing synoviocytes to proliferate into a characteristic fibroblast-like phenotype and justifying the choice of incorporating 0.8 mg ml^−1^ HA into the resultant microfluidic chip ECM.

To replicate RA disease conditions within the chip, we either introduced inflammatory cytokines IL-17 and TNF-*α*, both of which are heavily implicated in RA and have been shown to synergistically promote FLS invasion and the secretion of pro-inflammatory chemokines and MMPs [6, 58], or incorporated RA patient-derived synoviocytes (FLS-RAs) into the ECM. Characterization of these FLS-RAs in 2D culture (figure 1(g)) showed increased expression of CD90 (figure 1(h)) and decreased expression of PRG4 (figure 1(i)) compared to healthy FLSs through immunofluorescent staining. CD90 and PRG4 (lubricin) are predominantly expressed by synoviocytes in the subintima and intima, respectively, indicating that our synoviocytes display a subintimal phenotype [59, 60]. Additionally, our FLS-RAs in particular exhibit an aggressive, pro-inflammatory phenotype, characterized by enhanced proliferative potential and decreased secretion of lubricating molecules, as observed in vivo [61–63]. Furthermore, immunofluorescent staining of FLSs and FLS-RAs (figure 1(j)) showed secretion of HA typical of synoviocytes [64] as well as increased expression of actin (figure 1(k)) and CDH11 (figure 1(l)), both of which are associated with synoviocyte invasiveness in RA, confirming the diseased phenotype of patient-derived cells for use in our chips [65–67].

### 3.2. Synovium-on-chip recapitulates decreased lymphatic drainage and increased lymphatic permeability in RA via junctional remodeling

**Figure 2.**
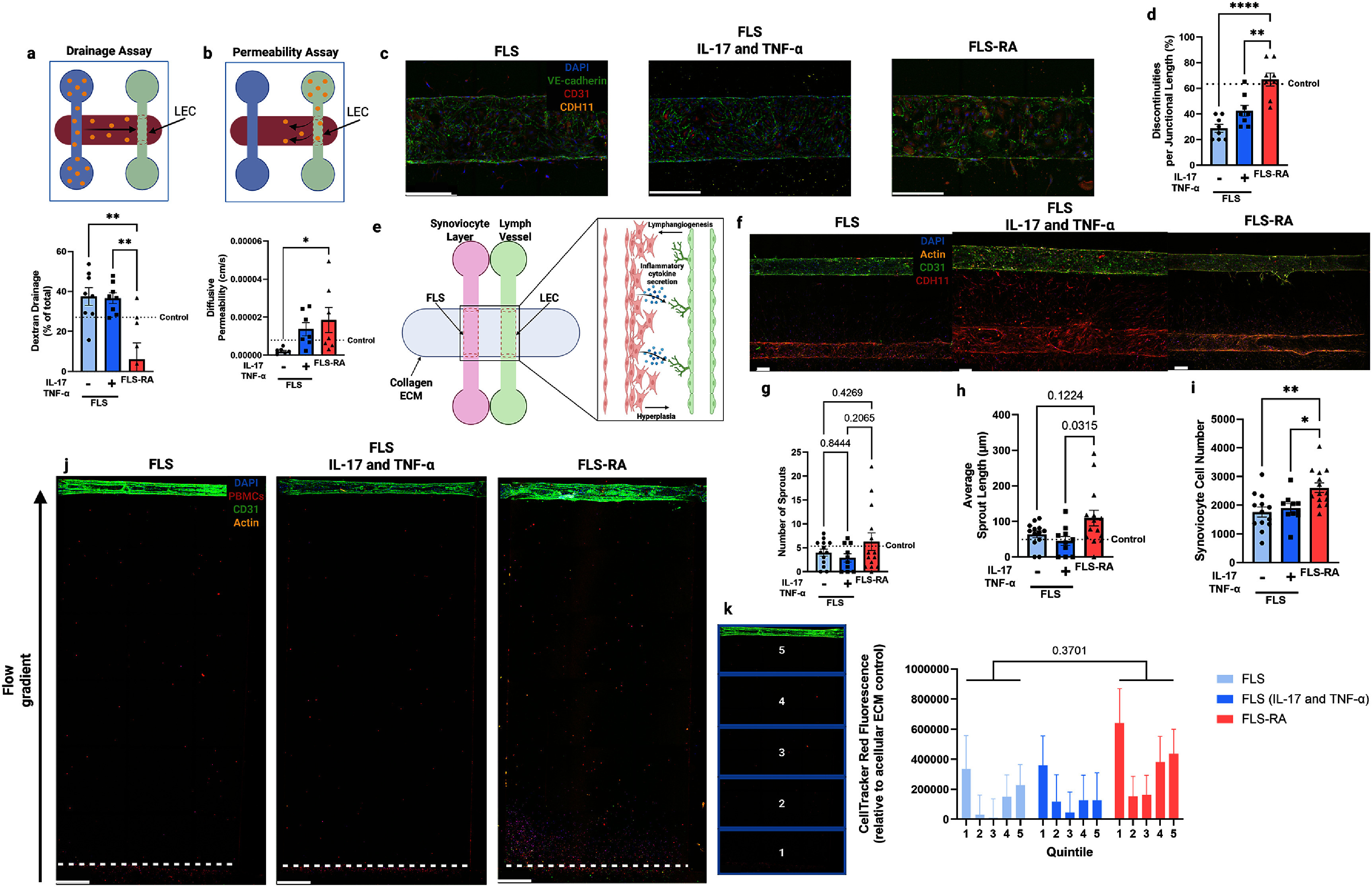
Microfluidic model exhibits impaired lymphatic drainage and permeability induced by RA. (a) In the drainage assay, fluorescent particles are added to the acellular channel to induce an interstitial fluid flow gradient towards the lymphatic vessel. Quantification of lymphatic drainage as a percentage of total fluorescent particles drained for chips. **p = 0.0020 (Control vs FLS+), **p = 0.0026 (FLS+ vs FLS-RA) (b) In the permeability assay, fluorescent particles are added to the reservoirs of the lymphatic vessel to create fluid hydrostatic pressure within the vessel lumen. Quantification of diffusive permeability for chips. *p = 0.0375 (c) Representative images of lymphatic vessels in synovium-on-chip stained with DAPI, VE-cadherin (adherens junctions), CD31 (endothelial cell marker), and CDH11 (FLS marker). Scale bar = 200 *μ*m. (d) Quantification of the percentage of discontinuities per total junctional area for chips. ****p < 0.0001, **p = 0.0016 (e) Design for lymphangiogenesis studies in synovium-on-chip, with an engineered lymphatic vessel embedded in collagen 1 matrix at 1 mm from the channel seeded with FLS to examine the paracrine effect of synoviocytes on LEC growth and behavior. (f) Representative images of lymphatic vessels and proximal FLS-seeded layer stained with DAPI, phalloidin (actin), CD31 (endothelial cell marker), and CDH11 (FLS marker). Scale bar = 200 *μ*m. Quantification of the (g) number of lymphangiogenic sprouts, (h) average sprout length, and (i) synoviocyte invasion indicated by cell number from the lymphangiogenesis assay. **p = 0.0024, *p = 0.0199; one-way ANOVA with Tukey’s HSD tests, *n* shown as individual data points for each group. Data are expressed as mean ± S.E.M. (j) Images from the immune cell migration assay in which fluorescently labeled PBMCs are added to the acellular channel to induce an interstitial fluid flow gradient towards the lymphatic vessel. Chips were stained with DAPI, CellTracker Red (PBMCs), CD31 (endothelial cell marker), and phalloidin (actin). Scale bar = 500 *μ*m. (k) To quantify PBMC migration, each image was divided into five equal segments, and the amount of CellTracker Red fluorescence in each quintile measured relative to chips with an acellular ECM control. Two-tailed paired Student *t*-tests, *n* = 6-8. Data are expressed as mean ± S.E.M.

To characterize lymphatic functionality within our synovial lymphatics microfluidic chip, we performed drainage and permeability assays. In the drainage assay (figure 2(a) top), fluorescent dextran particles are introduced into the acellular channel via a hydrostatic fluid gradient of cell media to model how efficiently the initial lymphatics uptake fluid and macromolecules from the interstitial tissue space. In the permeability assay (figure 2(b) top), the same fluorescent particles are added to the LEC channel reservoirs to model how well the lumen retains fluid and macromolecules for downstream transport to the lymph nodes [40, 68, 69]. The inclusion of RA patient-derived synoviocytes significantly decreased lymphatic drainage to nearly one-quarter of that observed in chips with healthy FLSs and even FLSs stimulated with inflammatory cytokines IL-17 and TNF-*α* (figure 2(a) bottom), in direct reflection of the stalled lymphatic drainage and edema seen during the collapsed, symptomatic phase of RA in vivo [19, 20, 22]. In parallel, FLS-RAs increased lymphatic permeability and leakiness within the synovium-on-chip to an even greater extent than with stimulation by a single inflammatory cytokine (figure 2(b) bottom). This aligns with the decreased transport capacity of the initial lymphatics during the collapsed phase, due to retained catabolic factors and inflammatory cytokines damaging LEC vessel integrity [70, 71]. To determine whether secreted factors or the interaction between FLSs and LECs contribute to this lymphatic dysfunction by altering vessel structure, immunofluorescent images of LVs in the chips were acquired (figure 2(c)). It was found that the presence of RA synoviocytes increased the appearance of LEC junctional disruptions (figure 2(d)), which is commonly correlated with increased lymphatic drainage and leakiness in disease [72, 73]. This remodeling, while similar to endothelial cell junctional ‘buttoning’ that normally allows for more efficient transcellular fluid and nutrient uptake into the lymphatics, is distinct in that it likely indicates dysfunction of the primary valves that prevent backflow and leakage [40], impairing retention and permeability as seen both in vivo with RA and our *in vitro* model. Furthermore, past studies suggest that endothelial cell gap formation and damage of connections to interstitial tissue may contribute to increased leakiness and edema in RA [74, 75].

### 3.3. RA synoviocytes induce lymphangiogenesis and exhibit hyperplastic growth in synovium-on-chip

To enable closer interaction between synoviocytes and LECs for lymphangiogenic studies, the synovium-on-chip model was modified by moving the FLSs to the previously acellular channel, 1 mm apart from the LEC channel (figure 2(e)). Inflammatory cytokines or stimulatory chemokines secreted by the adjacent FLS layer, similar to the thin intimal lining of the synovium, are thus able to exert paracrine effects on the engineered LV. Through immunofluorescent staining of lymphatics (CD31) and synoviocytes (CDH11) (figure 2(f)), it is observed that the number of lymphangiogenic sprouts was similar in healthy, cytokine-stimulated, and RA synoviocyte conditions (figure 2(g)), and that RA patient-derived synoviocytes induced greater lymphangiogenic sprout length (figure 2(h)) than healthy or cytokine-stimulated synoviocytes. This directly replicates the rapid lymphangiogenesis that occurs during the expansion phase of RA as a compensatory response to joint inflammation [76, 77], resulting in exacerbated proliferation of dysfunctional LVs, as seen in other inflammatory conditions such as Crohn’s disease [78, 79]. As seen in vivo with synovial pannus formation from FLS overgrowth [1], we also see increased synoviocyte proliferation with FLS-RAs, to the point of even invading the LEC channel (figure 2(i)).

### 3.4. Synovium-on-chip recapitulates immune cell retention and migration to inflamed interstitial tissue in RA

As autoreactive immune cell recruitment to the inflamed synovium as well as the immunomodulation by regulatory T cells is key to the pathogenesis of RA [80, 81], we introduced PBMCs labeled with CellTracker^TM^ Red to the acellular channel with a hydrostatic fluid gradient to examine subsequent migration through the synovial ECM, composed for this assay of FLSs within a fibrin-collagen composite hydrogel optimized for immune cell migration [52], to the LV (figure 2(j)). As determined by flow cytometry analysis (supplementary figure 1), the PBMC population consisted of 46.76% T cells, 14.81% B cells, and 10.9% monocytes, in accordance with immune cell populations isolated from human whole blood [82]. Dividing the ECM into five equal quintiles and examining PBMC movement (figure 2(k)), we observe that microfluidic chips with RA patient-derived synoviocytes showed an increased presence of PBMCs within the extracellular matrix, indicating increased immune cell recruitment but also retention within the inflamed synovium. This mimics the recruitment of autoreactive T cells and autoantibody production to the synovium in RA, as well as ‘B cell clogging’ of lymph nodes that occurs with recruitment of a unique subset of B cells [83–85].

### 3.5. RA synoviocytes overexpress CHI3L1, contributing to lymphatic dysfunction in synovium-on-chip and in vivo

**Figure 3.**
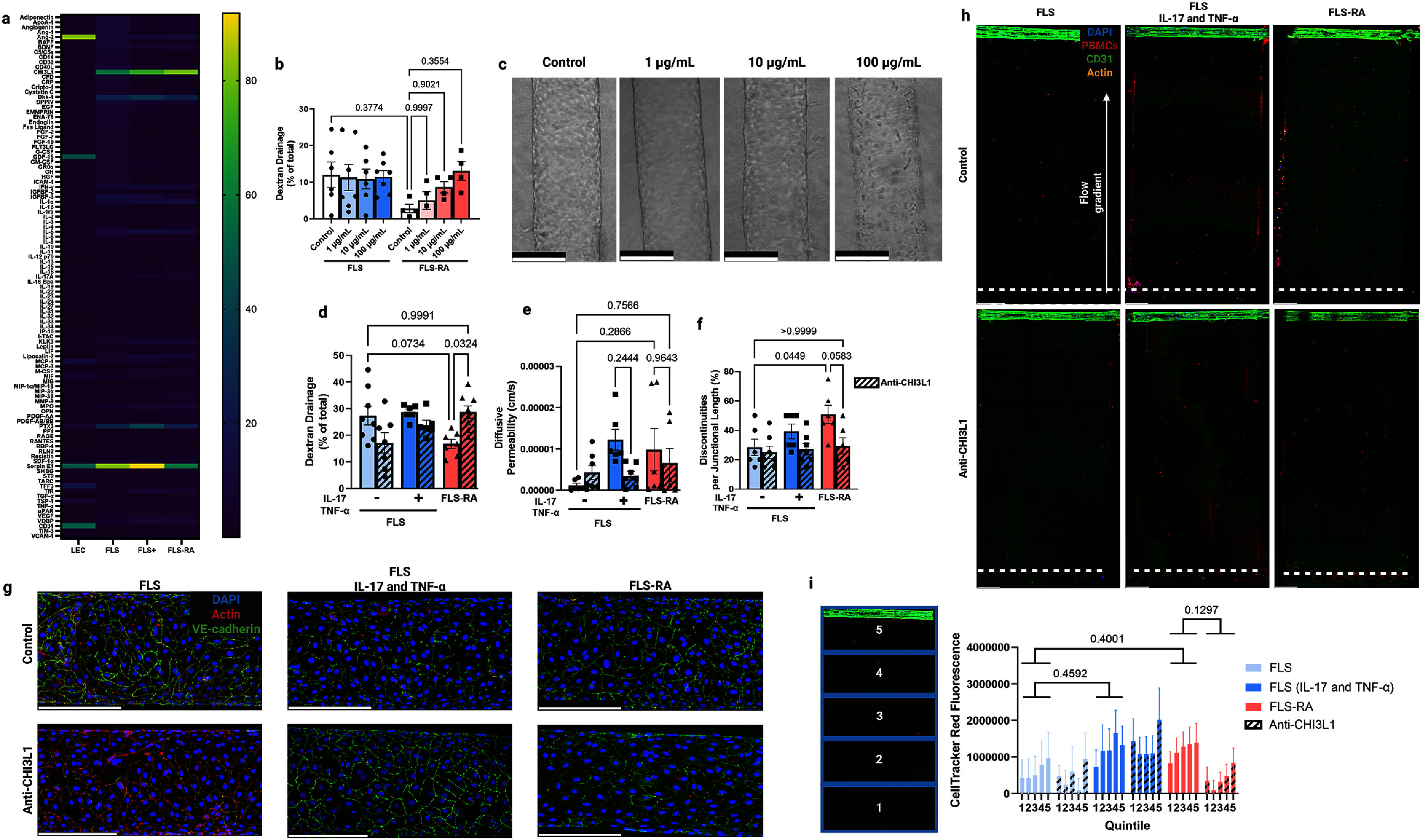
Lymphatic dysfunction of microfluidic chip under RA is attributed to overexpression of CHI3L1 and attenuated by CHI3L1 inhibition. (a) Heat map of relative expression of 105 human cytokines secreted from LECs, healthy FLS, FLS stimulated with inflammatory cytokines IL-17 and TNF-*α* (FLS+), and RA patient-derived FLS determined by membrane immunoassay. (b) Determination of appropriate CHI3L1 neutralizing antibody dose via quantification of drainage in microfluidic chips, as performed in a previous assay for both healthy and RA patient-derived FLS. (c) Representative images of engineered lymphatic vessels in microfluidic chips with healthy FLS treated with increasing concentration of CHI3L1 neutralizing antibody. Scale bar = 200 *μ*m. Quantification of (d) lymphatic drainage as a percentage of total fluorescent particles drained, (e) diffusive permeability, and (f) percentage of discontinuities per total junctional area for chips treated with anti-CHI3L1 neutralizing antibody. One-way ANOVA with Tukey’s HSD tests, *n* shown as individual data points for each group. Data are expressed as mean ± S.E.M. (g) Representative images of lymphatic vessels in synovium-on-chip treated with anti-CHI3L1 neutralizing antibody, stained with DAPI, phalloidin (actin), and VE-cadherin (adherens junctions). Scale bar = 200 *μ*m. (h) Images from immune cell migration assay for chips treated with anti-CHI3L1 neutralizing antibody. Chips were stained with DAPI, CellTracker Red (PBMCs), CD31 (endothelial cell marker), and phalloidin (actin). Scale bar = 500 *μ*m. (i) To quantify PBMC migration, each image was divided into five equal quintiles, and the amount of CellTracker Red fluorescence in each quintile was measured relative to the background signal. Two-tailed paired Student *t*-tests, *n* = 6-8. Data are expressed as mean ± S.E.M.

To elucidate the detailed molecular mechanism by which FLS-RAs induce impaired lymphatic drainage and unconstrained lymphangiogenesis, a multiple cytokine membrane-based sandwich ELISA immunoassay was used to screen for levels of 105 human cytokines from the media of LECs, healthy FLS, FLS stimulated with inflammatory cytokines IL-17 and TNF-*α*, and RA patient-derived FLS (figure 3(a)). Amongst the multiple targets screened from the cell secretomes, it was found that the presence of inflammatory cytokines or a unique phenotype of FLS-RAs increased expression of the CHI3L1 by 27% and 43% compared to healthy FLSs, respectively. CHI3L1 is a non-enzymatic chitinase-like protein that binds to structural ECM molecules chitin, heparin, and hyaluronic acid [86]. It is produced by a variety of cell types, including chondrocytes, synoviocytes, vascular smooth muscle cells, macrophages, neutrophils, endothelial cells, and cancer cells [87–89]. Currently, its secretion is known to be influenced by changes in ECM, miRNAs, growth factors, cytokines, stress, and drugs [90–92]. In the context of RA, CHI3L1 can promote synoviocyte growth through MAPK and Akt signaling [93] and exert a chemotactic effect on vascular endothelial cells during tissue injury and inflammation, thereby stimulating angiogenesis and lymphangiogenesis [94–96]. Most encouragingly, elevated CHI3L1 serum levels are seen in patients with RA and are associated with poor prognosis and joint destruction [97, 98], and CHI3L1 has even been identified as an autoantigen in RA [99, 100]. Thus, CHI3L1 secreted by inflamed synovial fibroblasts may lead to junctional disruption and impaired drainage observed in both the synovium-on-chip and human patients.

To assess a potential target protein for restoring lymphatic function, we repeated the previous drainage, permeability, and lymphangiogenesis experiments using a neutralizing antibody against human CHI3L1. The proper dosage of the neutralizing antibody was determined by treating healthy and RA patient-derived FLS-containing microfluidic chips with sequentially increasing inhibitor concentrations (1, 10, and 100 *μ*g ml^−1^) and performing a drainage assay. While the addition of the CHI3L1 inhibitor had no effect on chips with healthy FLS, drainage increased linearly for chips with RA patient-derived FLS, confirming the role of RA synoviocyte-secreted CHI3L1 in impairing lymphatic drainage (figure 3(b)). Brightfield images of LEC vessels in the chips, however, showed that higher concentrations, on the order of 100 *μ*g ml^−1^, of the CHI3L1 inhibitor dissociated vessels (figure 3(c)). Therefore, the dosage of 10 *μ*g ml^−1^ was chosen for the CHI3L1 inhibitor in ensuing experiments.

### 3.6. Inhibition of CHI3L1 in RA synoviocytes restores lymphatic drainage in synovium-on-chip

As expected from dosage-response studies, the inclusion of a CHI3L1-neutralizing antibody at 10 *μ*g ml^−1^ restored the decreased lymphatic drainage induced by FLS-RAs to levels nearly equivalent to those in healthy FLS microfluidic chips (figure 3(d)). In fact, inhibition of CHI3L1 in healthy FLS and in inflammatory cytokine-stimulated FLS chips showed an opposite trend, with decreased lymphatic drainage, albeit nonsignificantly, suggesting that CHI3L1 is specifically overexpressed by RA synoviocytes. Conversely, CHI3L1 inhibition had little effect on decreasing lymphatic permeability in chips with FLS-RAs, with the inhibitor treatment actually having a more pronounced effect at restoring vessel integrity to healthy conditions in the inflammatory cytokine-stimulated environment (figure 3(e)).

To visualize any lymphatic reorganization associated with CHI3L1, we examined immunofluorescent images of the synovial vessels in the chip stained with CHI3L1, and we examined immunofluorescent images of the vessels in the synovium chip stained with VE-cadherin (figure 3(g)). We found that the previously observed increase in junctional disruptions from RA synoviocytes was reversed by the addition of an anti-CHI3L1 antibody (figure 3(f)), thereby restoring vessel integrity and preventing impaired retention of fluid and macromolecules from junctional discontinuities. CHI3L1 has been shown to interact with IL-13R*α*2/TMEM219 to promote endothelial permeability and endothelial-mesenchymal transition in cancer, as well as with syndecan-1 and integrin *αvβ*5 to enhance tumor vascular permeability [101, 102], so its inhibition in RA may reverse the vessel leakiness and reduced uptake seen.

This effect of CHI3L1 inhibition to rescue lymphatic drainage extended to immune cells as well, for a repeated immune cell migration assay (figure 3(h)), revealed that anti-CHI3L1 neutralization decreased the recruitment of PBMCs to the synovial ECM, encouraging immune cell clearance and movement towards the LEC vessel under RA conditions (figure 3(i)). This could provide an avenue to resolve local RA inflammation by improving both fluid drainage to reduce edema and immune cell egress to remove cytotoxic CD8+ T cells and autoantibodies.

### 3.7. CHI3L1 protein impairs LV structure and function

**Figure 4.**
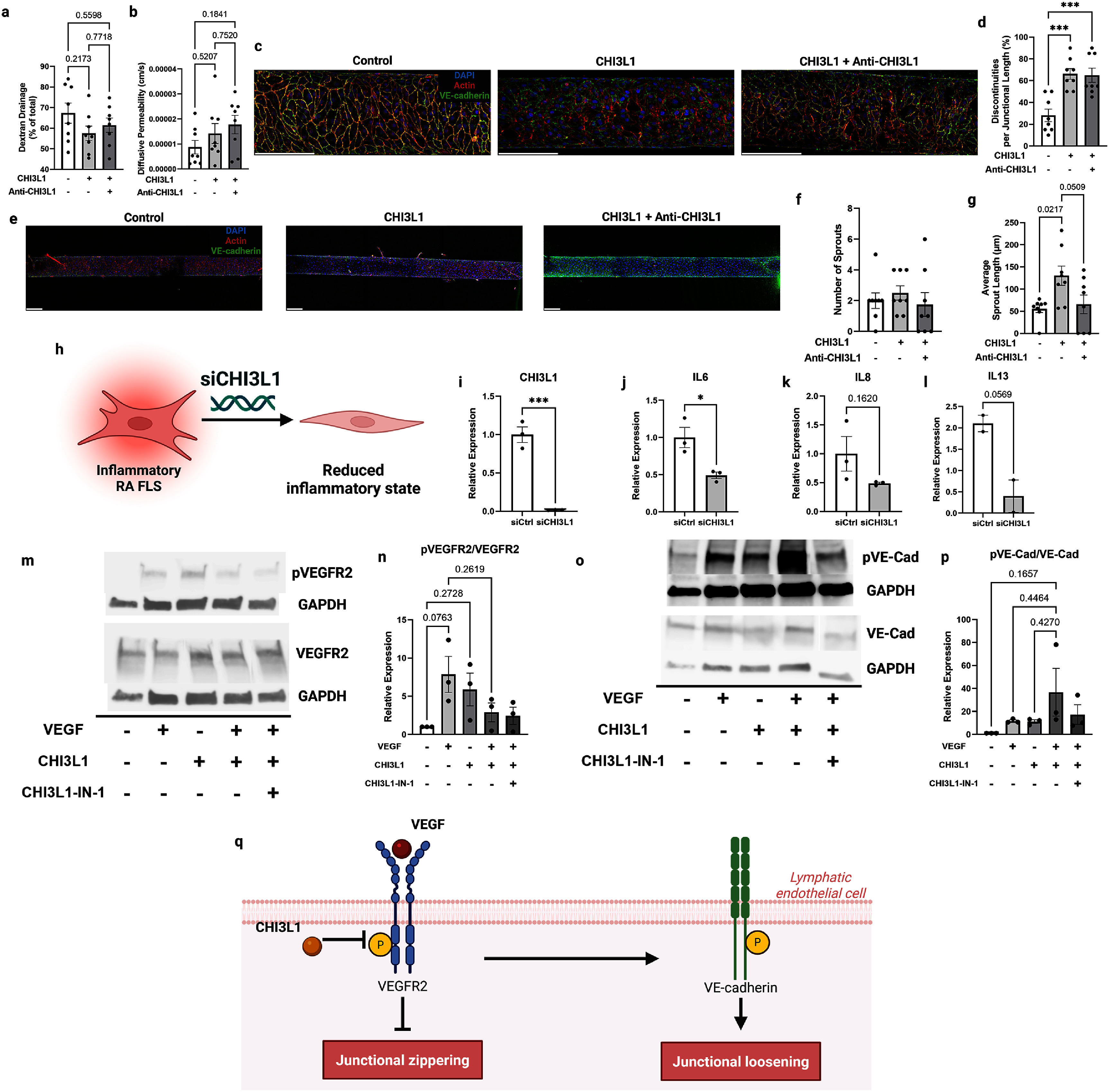
CHI3L1 impairs lymphatic drainage by altering VEGFR2 and VE-Cad phosphorylation in LECs. (a) Quantification of lymphatic drainage as a percentage of total fluorescent particles drained (b) and diffusive permeability for engineered lymphatic vessels in an acellular ECM, treating the vessel with soluble CHI3L1 protein or a combination of the protein and anti-CHI3L1 neutralizing antibody. (c) Representative images of lymphatic vessels treated with soluble CHI3L1, stained with DAPI, phalloidin (actin), and VE-cadherin (adherens junctions). Scale bar = 200 *μ*m. (d) Quantification of the percentage of discontinuities per total junctional area for chips in the previous section. ***p = 0.004 (Control vs CHI3L1), ***p = 0.005 (Control vs CHI3L1 + Anti-CHI3L1). (e) Representative images of lymphangiogenic sprouts in vessels treated with soluble CHI3L1 stained with DAPI, phalloidin (actin), and VE-cadherin (adherens junctions). Scale bar = 200 *μ*m. Quantification of the (f) number of lymphangiogenic sprouts and (g) average sprout length in the previous section. (h) Representation of siRNA-mediated knockdown of CHI3L1 expression in inflammatory RA patient-derived FLS and quantification from rt-qPCR analysis for relative gene expression of (i) CHI3L1, (j) IL6, (k) IL8, and (l) IL13 compared to control siRNA. *p = 0.0235, ***p = 0.0006; Two-tailed paired Student *t*-test, *n* shown as individual data points for each group. Data are expressed as mean ± S.E.M. Representative Western blots and quantification of (*m*)–(*n*) pVEGFR2 and (o)-(p) pVE-cadherin expression relative to total VEGFR2 and VE-cadherin expression, respectively, in LECs treated with combinations of VEGFA, CHI3L1, and CHI3L1-IN-1 inhibitor. One-way ANOVA with Tukey’s HSD tests, *n* shown as individual data points for each group. Data are expressed as mean ± S.E.M. (q) Schematic of the combinatorial effect of CHI3L1 with VEGFA on VEGFR2 and VE-cadherin in LECs. CHI3L1 secreted by RA synoviocytes, along with VEGFA, decreased phosphorylation of VEGFR2 and thus promoted button-like junctional discontinuities (left). In turn, CHI3L1 and VEGFA also increase phosphorylation of VE-cadherin and thus induce junctional loosening (right).

To determine that this rescue of lymphatic drainage is indeed due to CHI3L1 protein interaction with LECs, we repeated the drainage (figure 4(a)) and permeability (figure 4(b)) assays on a chip with a singular engineered LV and acellular ECM, treating the vessel with soluble CHI3L1 protein or a combination of the protein and anti-CHI3L1 neutralizing antibody. The addition of CHI3L1 modestly decreased drainage, whereas concurrent inhibition with a neutralizing antibody had no effect on drainage relative to the untreated control, implicating CHI3L1 in LEC reorganization that affects drainage, possibly in concert with other secreted factors from RA synoviocytes. CHI3L1 treatment also elicited a modest increase in permeability in engineered vessels, though the addition of a neutralizing antibody was less effective at resolving this, implicating lymphatic junctional disruption or damage resulting from CHI3L1 exposure. Upon examination of lymphatic junctional VE-cadherin staining (figure 4(c)), it is evident that CHI3L1 protein alone contributes to greater junctional disruption and loosening akin to button-like junctions compared to the untreated control (figure 4(d)). In accordance with observations from the synovium-on-chip in RA conditions and clinical observations [103], immunofluorescent staining of lymphangiogenic sprouting (figure 4(e)) shows that CHI3L1 encourages lymphangiogenesis of greater sprout length (figures 4(f) and (g)).

CHI3L1 also appears to be coupled with the upregulation of other combinatory inflammatory factors typically implicated in RA. With siRNA-mediated knockdown of CHI3L1 (figures 4(h) and (i)) in RA patient-derived synoviocytes, the gene expression of IL-6, IL-8, and IL-13, all of which are biomarkers of disease activity in RA [61], decreased (figures 4(j)–(l)). In particular, IL-6 has been shown to impair lymphatic endothelial vessel integrity and permeability by disrupting VE-cadherin [104, 105], similar to the junctional loosening observed in our model and supporting the interconnected role of CHI3L1 in mediating lymphatic dysfunction alongside other well-studied inflammatory factors.

### 3.8. CHI3L1 impairs lymphatic junctional organization by altering VEGFR2 and VE-cadherin phosphorylation in LECs

As CHI3L1 appears to have a significant impact on LEC junctional integrity and organization, we performed Western blot analysis to assess the relative expression of phosphorylated VEGFR2 (figure 4(m)) and VE-cadherin (figure 4(o)) to elucidate the specific downstream pathways by which CHI3L1 affects lymphatic junctions and thus synovial function. VEGFR2 is a well-known endothelial surface receptor responsible not only for lymphangiogenesis but also for activating downstream pathways such as PI3K/AKT, as VEGFR2 phosphorylation (pVEGFR2) is traditionally associated with junctional zippering [106–109]. VE-cadherin forms the basis of adherens junctions between LECs, and its phosphorylation has been implicated in junctional loosening and increased vessel permeability [110].

Encouragingly, we observed that the synergistic combination of VEGFA and CHI3L1 decreased pVEGFR2 compared to either factor alone, which could explain the loss of junctional zippering and the increase in button-like junctional disruptions observed in RA conditions on our microfluidic chip (figure 4(n)). VEGFA is a major stimulator of angiogenesis and vascular permeability through upregulation of VEGFR2 phosphorylation [111, 112], and CHI3L1 has been shown to promote angiogenesis in the tumor microenvironment [113, 114]. Both factors are observed to increase VEGFR2 phosphorylation individually in our study, consistent with the literature. The reason for the downregulation of pVEGFR2 in the combinatorial addition of VEGFA and CHI3L1, most representative of the cytokine environment within the synovium-on-chip in RA conditions, is still unknown but is worth further investigation, as the interaction between VEGFA and CHI3L1 has been previously shown to modulate each other’s expression through both positive and negative regulation [115].

Furthermore, the combination of VEGFA and CHI3L1 increased VE-cadherin phosphorylation in LECs compared with either factor alone (figure 4(p)). CHI3L1 has indeed been shown to induce upregulation of pVE-cadherin and contribute to junctional loosening and loss of vessel integrity [116], which aligns with the increased permeability and impaired drainage capacity observed in the synovium-on-chip model under RA conditions. We also observed increased activation of tyrosine kinase Src in LECs with VEGFA and CHI3L1 (supplementary figure 2), providing a potential linkage between altered phosphorylation of VEGFR2 and VE-cadherin, as Src in endothelial cells couples a VEGFA-induced signaling axis that results in VEGFR2 activation and VE-cadherin phosphorylation and fragmentation [108, 117, 118]. Thus, increased levels of CHI3L1 secreted by RA synoviocytes promote button-like junctional discontinuities by downregulating pVEGFR2 and impair junctional integrity by upregulating pVE-cadherin, contributing to decreased drainage and increased permeability within the synovial lymphatics (figure 4(q)).

### 3.9. CIA mouse model verifies *in vitro* lymphatic dysfunction in RA and shows clinical improvement with CHI3L1 inhibition

**Figure 5.**
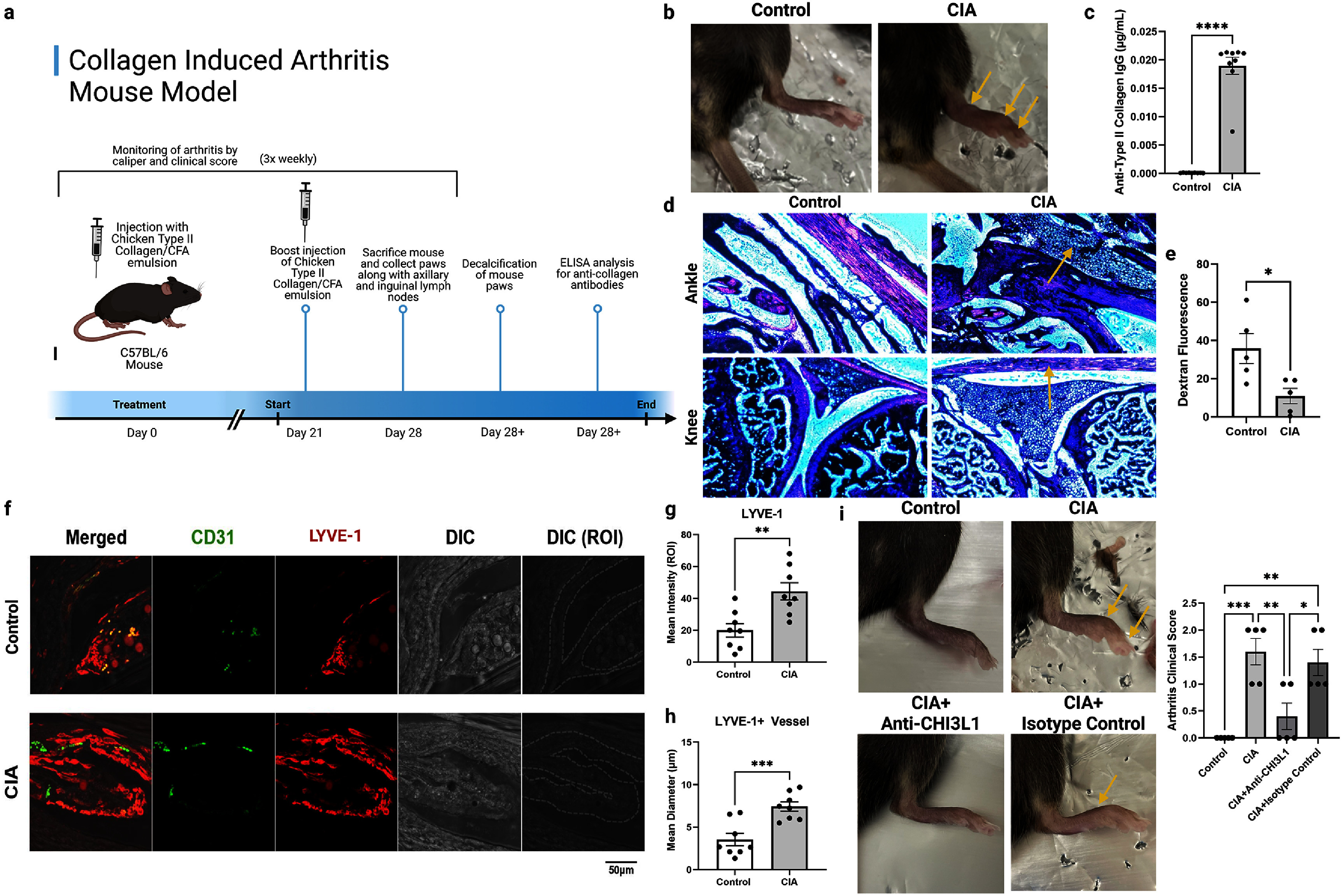
Collagen-induced arthritis mouse model verifies lymphatic dysfunction in RA and improvement with CHI3L1 inhibition. (a) Timeline of protocol for collagen-induced arthritis (CIA) model in C57BL/6 mice, which involves injection of a chicken type II collagen/Complete Freund’s Adjuvant (CFA) emulsion to promote production of autoantibodies. (b) Representative images of mouse back paws in control and CIA groups. Arrows show redness and swelling of (from left to right) carpal and torsal joint, metacarpophalangeal joint, and interphalangeal joint. (c) Quantification of production of anti-type II collagen IgG autoantibodies produced in control and CIA groups. ****p < 0.0001 (d) Representative images of hematoxylin and eosin staining of ankle and knee joints of mice in control and CIA groups. Top arrow shows immune cell infiltration; bottom arrow shows synovial lining hyperplasia. (e) Quantification of dextran fluorescence in popliteal lymph nodes after footpad injection for control and CIA groups 8 weeks after initial CIA inoculation. *p = 0.0222 (f) Representative immunofluorescent images of knee joint synovial membrane in control and CIA mice stained for CD31 (endothelial cell marker) and LYVE-1 (lymphatic endothelial cell marker). Dashed lines indicate ROI of synovium selected for image analysis. (g) Quantification of mean intensity of LYVE-1 signal and (h) mean diameter of LYVE-1+ vessels in synovial membrane for control and CIA mice. **p = 0.0030, ***p = 0.0008; Two-tailed paired Student *t*-test, *n* shown as individual data points for each group. Data are expressed as mean ± S.E.M. (i) Representative images of mouse back paws and clinical arthritis score for mice in control, CIA, CIA with anti-CHI3L1 neutralizing antibody treatment, and CIA with IgG isotype control treatment 5 weeks after initial CIA inoculation. Arrows show swelling and deformation of the indicated joints. *p = 0.0198, **p = 0.0013 (Control vs CIA + Isotype Control), **p = 0.0051 (CIA vs CIA + Isotype Control), ***p = 0.0004; one-way ANOVA with Tukey’s HSD tests, *n* shown as individual data points for each group. Data are expressed as mean ± S.E.M.

To verify our synovium-on-chip as a viable substitute for synovial lymphatics in animal models, we used the CIA mouse model (figure 5(a)) to induce autoantibody production and joint destruction in RA [55]. After 6 weeks of initial injection of chicken type II collagen and CFA, mice developed inflammation in one or more joints of the paw with visible swelling and redness (figure 5(b)). This was supported by the presence of anti-type II collagen autoantibodies in the serum of CIA mice (figure 5(c)) as well as inflammatory immune cell infiltration (figure 5(d), top) and synovial lining hyperplasia (figure 5(d), bottom). As a quantitative measure of lymphatic drainage and function, fluorescent dextran, which, due to its size of 70 kDa is primarily uptaken by the lymphatic capillaries, was injected into the footpad of control and CIA mice, and the subsequent fluorescence in the draining popliteal lymph nodes was measured. At 8 weeks post-inoculation, CIA mice exhibited decreased drainage compared with controls (figure 5(e)), reflecting the symptomatic collapsed phase of RA and the impaired drainage observed in our microfluidic model. Additionally, immunofluorescent staining of mouse knee joints (figure 5(f)) showed increased LYVE-1 signal (figure 5(g)) and larger LV diameters (figure 5(h)) in CIA mice, reflecting the heightened lymphangiogenesis observed clinically and in our *in vitro* model. Promisingly, treatment with a neutralizing antibody specific for mouse CHI3L1 even ameliorated the clinical arthritis score in CIA mice (figure 5(i)), indicating resolution of lymphedema and swelling, consistent with proper lymphatic function as seen *in vitro*.

## 4. Discussion

To examine the modulatory effects of FLS on lymphatic structure and function in RA, we created a synovium-on-chip to model the synovial lymphatics *in vitro*, which, to our knowledge, is the first microfluidic device to replicate the synovial lymphatic microenvironment (figures 1(a)–(d)). To provide a biomimetic synovium ECM, we optimized a collagen hydrogel with hyaluronic acid and fibronectin at physiological concentrations for co-culture of LECs and FLSs (figures 1(e) and (f)), which allowed appropriate vascular lumen formation and an elongated fibroblast phenotype. For the introduction of RA inflammatory conditions, we used a mixture of synergistic pro-inflammatory cytokines, IL-17 and TNF-*α*, or incorporated RA patient-derived synoviocytes, which we confirmed to show increased CDH11 and CD90 expression and decreased PRG4 expression (figures 1(g)–(l)), as typical of a pro-invasive, hyperplastic phenotype.

Our synovium-on-chip exhibited decreased lymphatic drainage (figure 2(a)) and increased permeability (figure 2(b)) with the inclusion of RA synoviocytes, due to an increased number of junctional discontinuities (figures 2(c) and (d)) that caused vessel leakiness and damage, mimicking the collapsed phase of the synovial lymphatics observed clinically during disease progression. RA synoviocytes also induced lymphangiogenesis and synovial hyperplasia in our chip (figures 2(e)–(i)), as seen in the early stages of RA, as well as immune cell retention and stasis in the interstitial tissue (figures 2(j) and (k)), which exacerbates the inflammatory microenvironment.

Through multi-cytokine ELISA analysis (figure 3(a)), we found a target protein secreted in excess by RA synoviocytes capable of inducing lymphatic dysfunction, CHI3L1, which when inhibited in RA-FLS chips restored lymphatic drainage to healthy levels (figure 3(d)) by reversal of RA junctional loosening (figures 3(f) and (g)). This effect also improved immune cell clearance and drainage from the synovial ECM (figures 3(h) and (i)).

We confirmed that the CHI3L1 protein could induce decreased drainage and increased permeability in LVs (figures 4(a) and (b)) via signaling for junctional disruption and lymphangiogenesis in lymphatic endothelial cells (figures 4(c)–(g)). Molecular-level gene analysis also revealed that knockdown of CHI3L1 in inflamed synoviocytes led to downregulation of inflammatory interleukins IL-6, IL-8, and IL-13 (figures 4(h)–(l)). With preliminary analysis of downstream pathways involved in junctional remodeling, we identified VEGFR2 and VE-cadherin as potential targets for the CHI3L1 mechanism of action in LVs via alteration of phosphorylation (figures 4(m)–(q)).

Using a validated CIA mouse model (figures 5(a)–(h)), we observed decreased lymphatic drainage in the collapsed phase of RA. Additionally, CHI3L1 inhibition in the CIA model improved RA symptoms in mice, suggesting a potential role in resolving lymphatic inflammation (figure 5(i)).

The hypothesis that CHI3L1 leads to lymphatic loosening is in line with previous studies that have found inflammatory cytokine stimulation impairs LEC barrier function and increases diffusive permeability via reduced expression of VE-cadherin [73]. However, it is not clear how specifically junctional discontinuities lead to both decreased drainage and increased permeability, as studies in other inflammatory diseases, such as Crohn’s disease or viral infection, show that initially permeable lymphatics transition to tight, zipper-like junctions that reduce fluid uptake [119–121]. Additionally, since junctional zippering is commonly associated with reduced drainage rather than the buttoning seen in our model [72, 120] the discontinuities within our model may represent a different junctional phenotype more akin to disruptions of intact adherens junctions. Possibly, a balance between adherens junctional integrity and primary valve function controls lymphatic drainage, as both are necessary for the one-way transport of fluid and macromolecules. A vessel abundant in button-like junctions may be more permeable. Still, if the anchoring filaments and other primary valve components are not working in concert, the valve may be more prone to leaks. This concept can be explored further. Additionally, although our microfluidic model used primary dermal LECs, synovial-specific lymphatics may possess unique phenotypic characteristics that make their behavior distinct from that of other tissues.

Regarding the therapeutic effects of CHI3L1 inhibition, this treatment shows promise in restoring lymphatic drainage but not necessarily in preventing synovial fibroblast hyperplasia and unregulated lymphangiogenesis in RA. Increased lymphangiogenesis in early RA is considered a compensatory response to joint inflammation; in fact, inhibiting lymphangiogenesis during arthritic progression exacerbates inflammation [77]. Given that the late stages of RA witness both lymphangiogenesis and a decrease in the number of LVs, encouraging lymphangiogenesis has been a popular strategy for RA. Therapies promoting lymphangiogenesis in RA by stimulating the VEGF-C/VEGFR-3 pathway have been shown to reduce synovitis, bone erosion, cartilage loss, and the number of infiltrating macrophages [122]. Even so, the timing of lymphangiogenic treatment is critical for disease mitigation, as lymphangiogenic modulation can inadvertently saturate lymph node surveillance, thereby increasing systemic exposure to unfiltered pathogens and inflammatory cytokines [123]. Within our microfluidic model, we observed both decreased lymphatic drainage and increased lymphangiogenesis in response to RA synoviocytes, suggesting that this inflammation-associated lymphangiogenesis may produce dysfunctional LVs prone to leakage and backflow.

Additionally, our microfluidic system may not fully model the complexity of the synovial lymphatics due to several factors. The native synovial ECM is heterogeneous and composed of multiple tissue types, including fibrous, areolar, and adipose [57]. Previous studies have utilized the cell-adherence and encapsulation properties of Matrigel [124] to form a biomimetic ECM, but this material suffers from inherent batch-to-batch variability and limited physiological relevance due to its tumor-derived origin. Other methods have involved cell-laden decellularized extracellular matrices of the synovium or meniscus [125], which preserve the native tissue architecture, but can be limited by immunogenicity and loss of structural properties. Our study optimized a collagen 1 hydrogel that is both physiologically relevant and ideal for *in vitro* culture of vasculature, incorporating hyaluronic acid to co-culture LECs and synoviocytes, but could benefit from other technologies, such as bioprinting and synthetic hydrogels, to create a more biomimetic environment. MLSs also populate the synovial ECM, and the intimal layer is buffered by a thin MLS layer [57]. The contribution of these synoviocytes, as well as other tissue-resident cells, may provide other chemokine and mechanical signaling to the lymphatics. We also assumed that RA patient-derived synoviocytes were sufficient on their own to mimic the RA environment. Still, in reality, multiple inflammatory cytokines and stimuli, including IL-6, IL-1, and GM-CSF [126], are closely interlinked in RA pathophysiology and may also contribute to lymphatic dysfunction. Furthermore, our synovium-on-chip model of the lymphatic capillaries within the synovial tissue shows that the most pronounced LV expansion and collapse occur downstream in the collecting LVs, which are lined by LMCs [19, 127]. Future iterations of this model could incorporate LMCs to fully observe the impaired contractile ability seen in RA.

The identification of CHI3L1 as a potential target for lymphatic restoration in RA warrants further exploration of downstream targets and precise molecular mechanisms of LEC remodeling, particularly given that VEGFR2 and VE-cadherin phosphorylation may be involved through mediation by Src, which has historically been studied in blood endothelial cells rather than lymphatics [108, 117]. CHI3L1 can bind to multiple cell receptors, including IL-13R*α*2/TMEM219 and syndecan-1/integrin *αvβ*5, thereby preventing cell death via the ERK1/2 and AKT pathways, promoting cell invasion, and increasing vascular permeability via the *β*-catenin, FAK, and ERK1/2 (MAPK) pathways [128]. Given that RA synoviocytes and secreted CHI3L1 impair LV integrity and promote LEC growth during lymphangiogenesis, these pathways could be involved in lymphatic dysfunction in our model and in vivo. As we demonstrate that CHI3L1 overexpression is also linked to the regulation of other inflammatory factors such as IL-6 and IL-8, future studies can incorporate CHI3L1 knocked-down synoviocytes within our microfluidic model to further establish the target’s role in lymphatic dysfunction. In addition, in-depth, long-term studies of CHI3L1 inhibition in the CIA mouse model could assess the effects on lymphatic drainage using fluorescent dye tracking to the lymph nodes, staining of the synovial-draining lymph nodes to examine specific immune cell subsets, and cell junctional changes within the synovial LECs.

## 5. Conclusions

The role of lymphatics in the pathogenesis of RA is a critically understudied area that can be elucidated through novel, physiologically relevant microfluidic *in vitro* modeling. To this end, we create a synovium-on-chip model with associated lymphatics. Through functional testing, we can replicate increased lymphatic permeability and decreased drainage in RA inflammation due to endothelial junctional disruption, as seen in vivo. We also demonstrate, through a proof of concept identifying a CHI3L1-VEGFR2/VE-cadherin signaling axis in inflammatory RA synoviocytes, that our device can be used for mechanistic studies of lymphatic dysfunction, informing lymphatic-targeting therapeutics. Our synovium-on-chip model, as the first of its kind, can provide a physiologically accurate representation of lymphatic drainage and activity under disease conditions, informing tissue engineering, drug testing, and high-throughput screening for rheumatoid arthritis.

## Data Availability

All data that support the findings of this study are included within the article (and any supplementary files). Supplementary Material   available at: https://doi.org/10.1088/1758-5090/ae9348/data1.
